# Supplementary material for: Garcinol Attenuates Lipoprotein(a)-Induced Oxidative Stress and Inflammatory Cytokine Production in Ventricular Cardiomyocyte through α7-Nicotinic Acetylcholine Receptor-Mediated Inhibition of the p38 MAPK and NF-κB Signaling Pathways
Source: Antioxidants (Basel). 2021 Mar 16;10(3):461. doi: 10.3390/antiox10030461 (PMC8000018; doi:10.3390/antiox10030461)
Supplement: Supplementary file 1 [file antioxidants-10-00461-s001.pdf]

Supplementary Information

Garcinol attenuates lipoprotein(a)-induced oxidative stress and inflammatory cytokine production in ventricular cardiomyocyte through α7-nicotinic acetylcholine receptor-mediated inhibition of the p38 MAPK and NF-κB signaling pathways

Disclosed by Nen-Chung Chang [1,2,3,‡], Chi-Tai Yeh [4,5,‡], Yen-Kuang Lin [6,7,8], Kuang-Tai Kuo [9,10], Iat-Hang Fong [4,5], Nicholas G. Kounis [11], Patrick Hu [12,13] and Ming-Yow Hung [2,3,14,*]

[1] Division of Cardiology, Department of Internal Medicine, Taipei Medical University Hospital, Taipei 110, Taiwan

[2] Division of Cardiology, Department of Internal Medicine, School of Medicine, College of Medicine,

Taipei Medical University, Taipei 110, Taiwan; ncchang@tmu.edu.tw

[3] Taipei Heart Institute, Taipei Medical University, Taipei 110, Taiwan

[4] Department of Medical Research and Education, Taipei Medical University—Shuang Ho Hospital, New Taipei City **23561**, Taiwan; ctyeh@s.tmu.edu.tw (C.-T.Y.); impossiblewasnothing@hotmail.com (I.-H.F.)

[5] Department of Medical Laboratory Science and Biotechnology, Yuanpei University of Medical Technology, Hsinchu City 30015, Taiwan.

[6] Biostatistics Center, Office of Data Science, Taipei Medical University, Taipei **110**, Taiwan.

[7] Graduate Institute of Data Science, Taipei Medical University, Taipei **110**, Taiwan.

[8] Research Center of Big Data, College of Management, Taipei Medical University, Taipei **110**, Taiwan; robbinlin@tmu.edu.tw

[9] Division of Thoracic Surgery, Department of Surgery, Shuang Ho Hospital, Taipei Medical University, New Taipei City **23561**, Taiwan; doc2738h@gmail.com

[10] Division of Thoracic Surgery, Department of Surgery, School of Medicine, College of Medicine, Taipei Medical University, Taipei **110**, Taiwan

[11] Department of Internal Medicine, Division of Cardiology, University of Patras Medical School, Patras **26221**, Greece; ngkounis@otenet.gr

[12] University of California, Riverside, Riverside, CA **92521**, USA; patrick.p.hu@gmail.com

[13] Department of Cardiology, Riverside Medical Clinic, Riverside, CA **92506**, USA

[14] Division of Cardiology, Department of Internal Medicine, Shuang Ho Hospital, Taipei Medical University, New Taipei City **23561**, Taiwan

***** Correspondence: myhung6@ms77.hinet.net ; Tel.: +886 2 22490088

‡ They contributed equally to this work.

# Drs Chi-Tai Yeh, and Nen-Chung Chang contributed equally.

***Corresponding authors:** Ming-Yow Hung, MD, PhD, FAHA. Division of Cardiology, Department of Internal Medicine, Shuang Ho Hospital, Taipei Medical University, No.291, Zhongzheng Rd., Zhonghe District, New Taipei City, 23561, Taiwan. Tel.: +886 2 22490088; Fax: +886 2 82622010; E-mail: myhung6@ms77.hinet.net

Running Title: Lipoprotein(a) medicates CAS through α7-nAChR/p38MAPK signaling.

Supplementary Table S1. Primer sequence in this study

Gene		sequence
α7-nAChR	forward	GCCAATGACTCGCAACCACTC
	reverse	CCAGCGTACATCGATGTAGCA
IL-6	forward	AGACAGCCACTCACCTCTTCAG
	reverse	TTCTGCCAGTGCCTCTTTGCTG
TNF-α	forward	CTCTTCTGCCTGCTGCACTTTG
	reverse	ATGGGCTACAGGCTTGTCACTC
CRP	forward	TCGACGCGTGGAAAGAG
	reverse	TAGAATCAGATTATCCTGAC
NFκB	forward	GCAGCACTACTTCTTGACCACC
	reverse	TCTGCTCCTGAGCATTGACGTC

Supplementary Table S2. Western blot antibodies sheet.

No.	Target	Dilution	Source	
1	α7-nAChR	1:500	ab216485	abcam
2	CaMKII	1:1000	#50049	Cell signaling
3	p-CaMKII	1:1000	#12716	Cell signaling
4	Erk1/2	1:5000	#4695	Cell signaling
5	p-Erk1/2	1:5000	#4370	Cell signaling
6	p38 MAPK	1:1000	#8690	Cell signaling
7	p-p38 MAPK	1:1000	#4511	Cell signaling
8	GSK-3β	1:2000	#12456	Cell signaling
9	p-GSK-3β	1:2000	#5558	Cell signaling
10	IL-6	1:2000	ab6672	abcam
11	TNFA	1:5000	ab92324	abcam
12	CRP	1:5000	ab185558	abcam
13	NF-kB	1:1000	ab16502	abcam
14	IGF-IIR	1:1000	sc-136321	Santa Cruz
15	HSF1	1:1000	sc-17757	Santa Cruz
16	caspase-3	1:1000	sc-7272	Santa Cruz
17	VCAM-1	1:1000	#39036	Cell signaling
18	ICAM-1	1:1000	#4915	Cell signaling
19	E-Selectin	1:1000	sc-137054	Santa Cruz
20	p-MBS	1:1000	#3040	Cell signaling
21	t-MBS	1:1000	#2634	Cell signaling

22	RhoA	1:1000	#2117	Cell signaling
23	RhoA-GTP	1:1000	#8820	Cell signaling
24	ROCK1	1:1000	#4035	Cell signaling
25	ROCK2	1:1000	#9029	Cell signaling
26	β-actin	1:10000	66009-1-Ig	PROTEINTECH

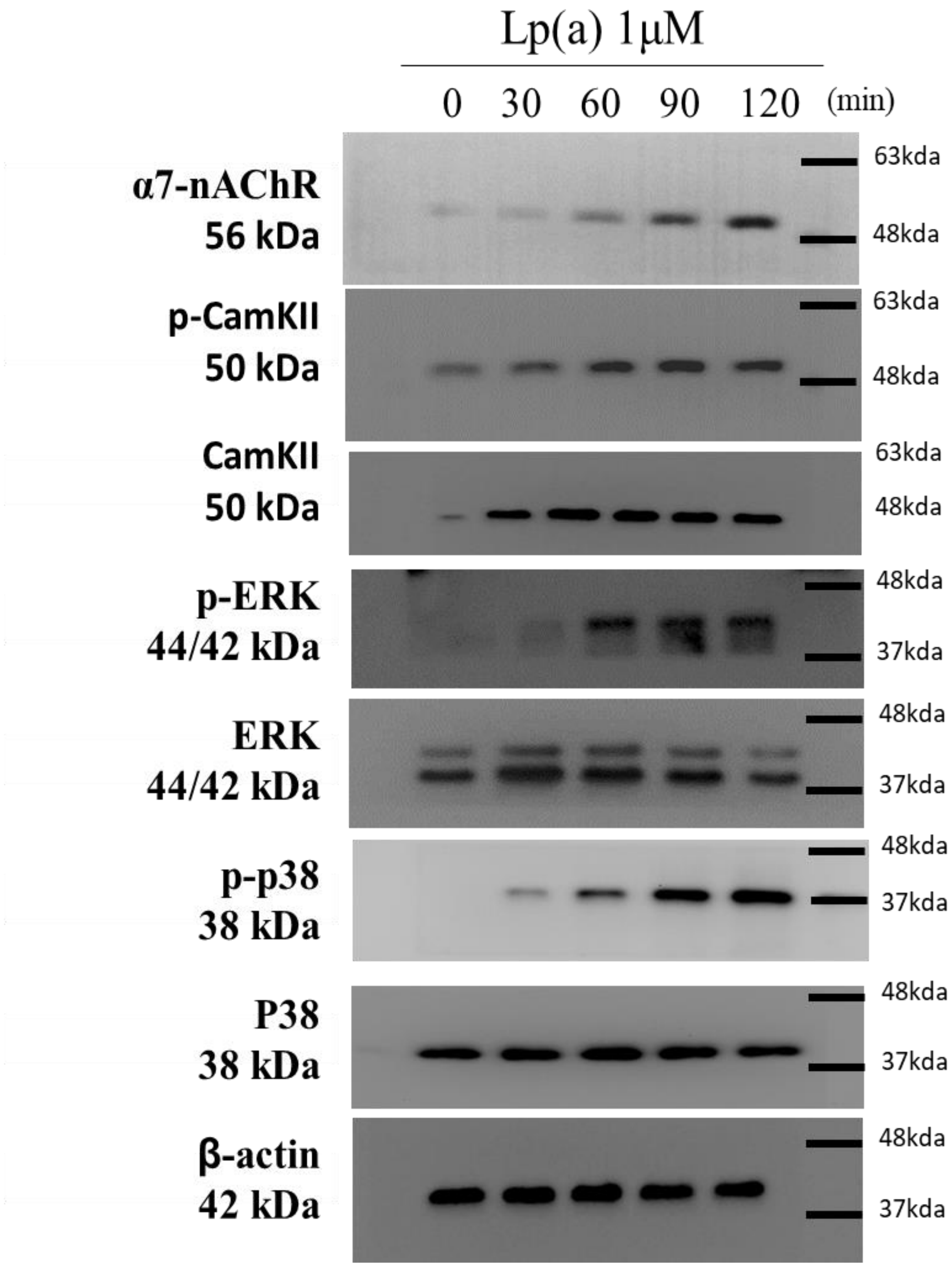

Supplementary Figure S1. Full-size blots of Figure 1F

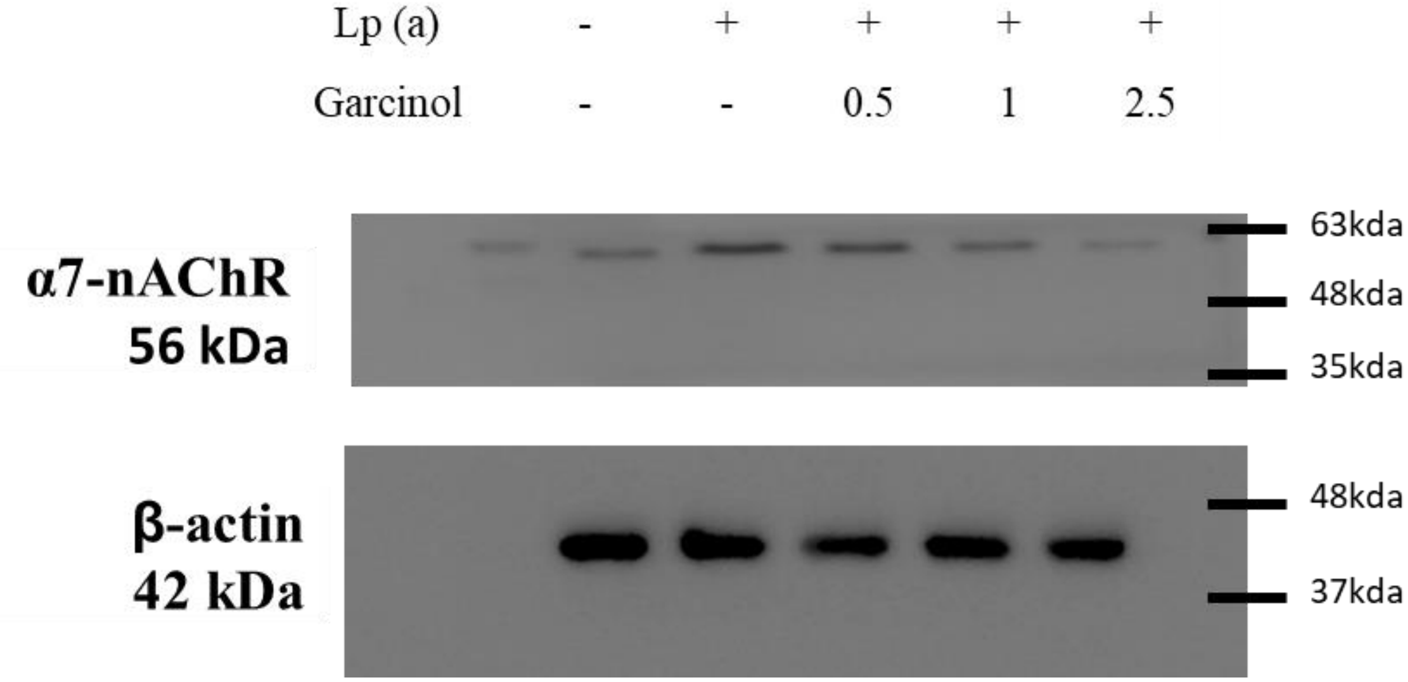

Supplementary Figure S2. Full-size blots of Figure 2F

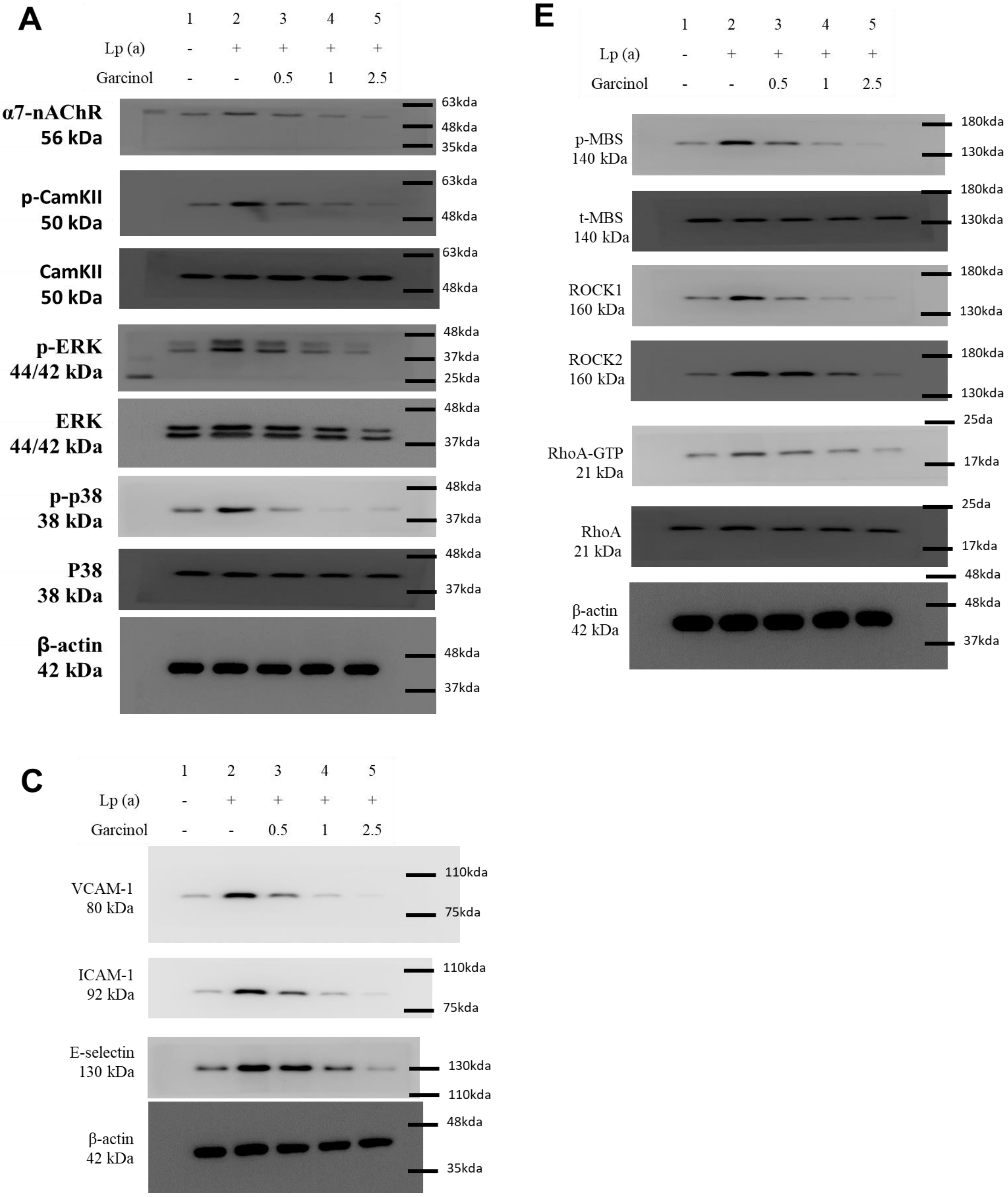

Supplementary Figure S3. Full-size blots of Figure 2A, 3C and 3E

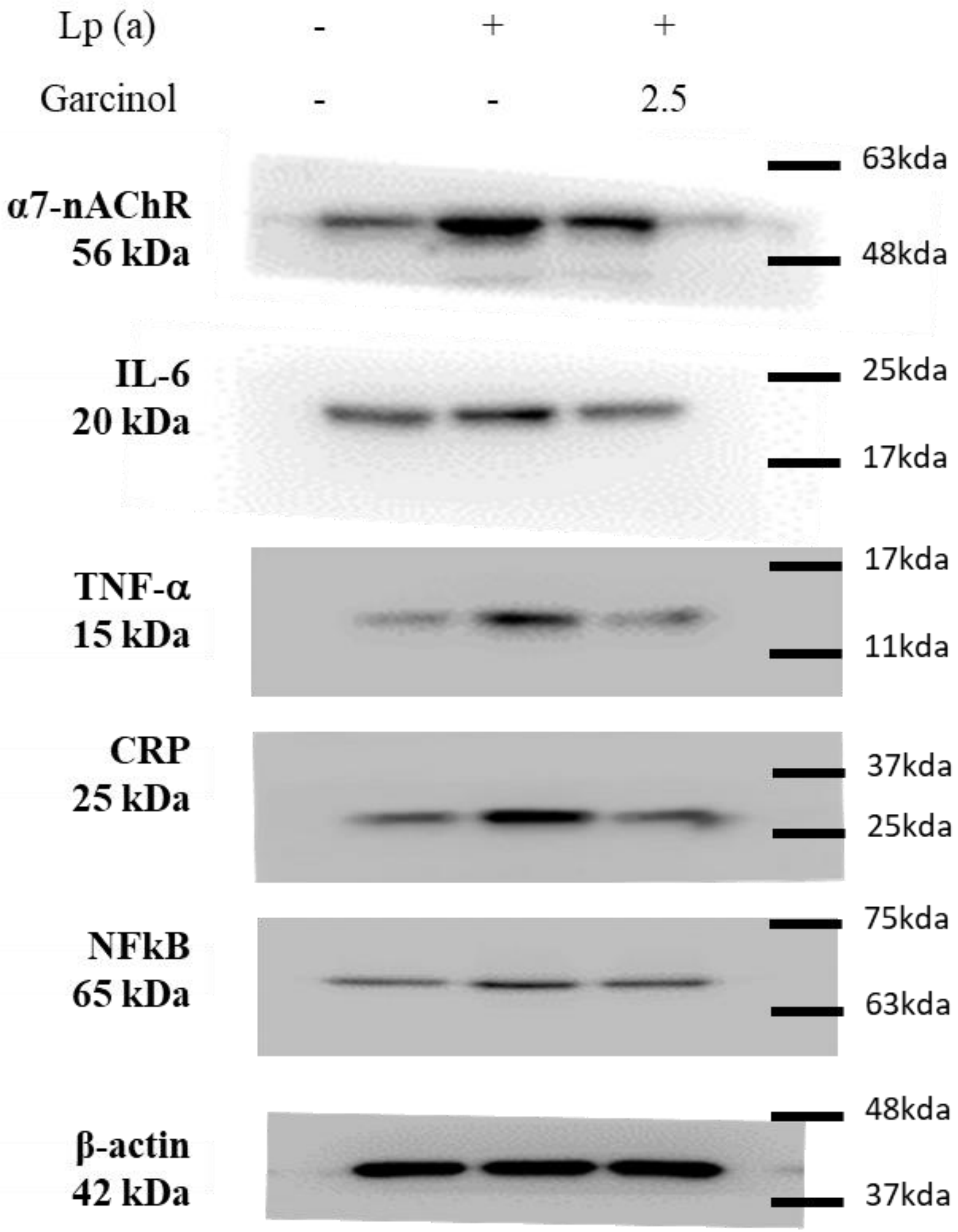

Supplementary Figure S4. Full-size blots of Figure 4B

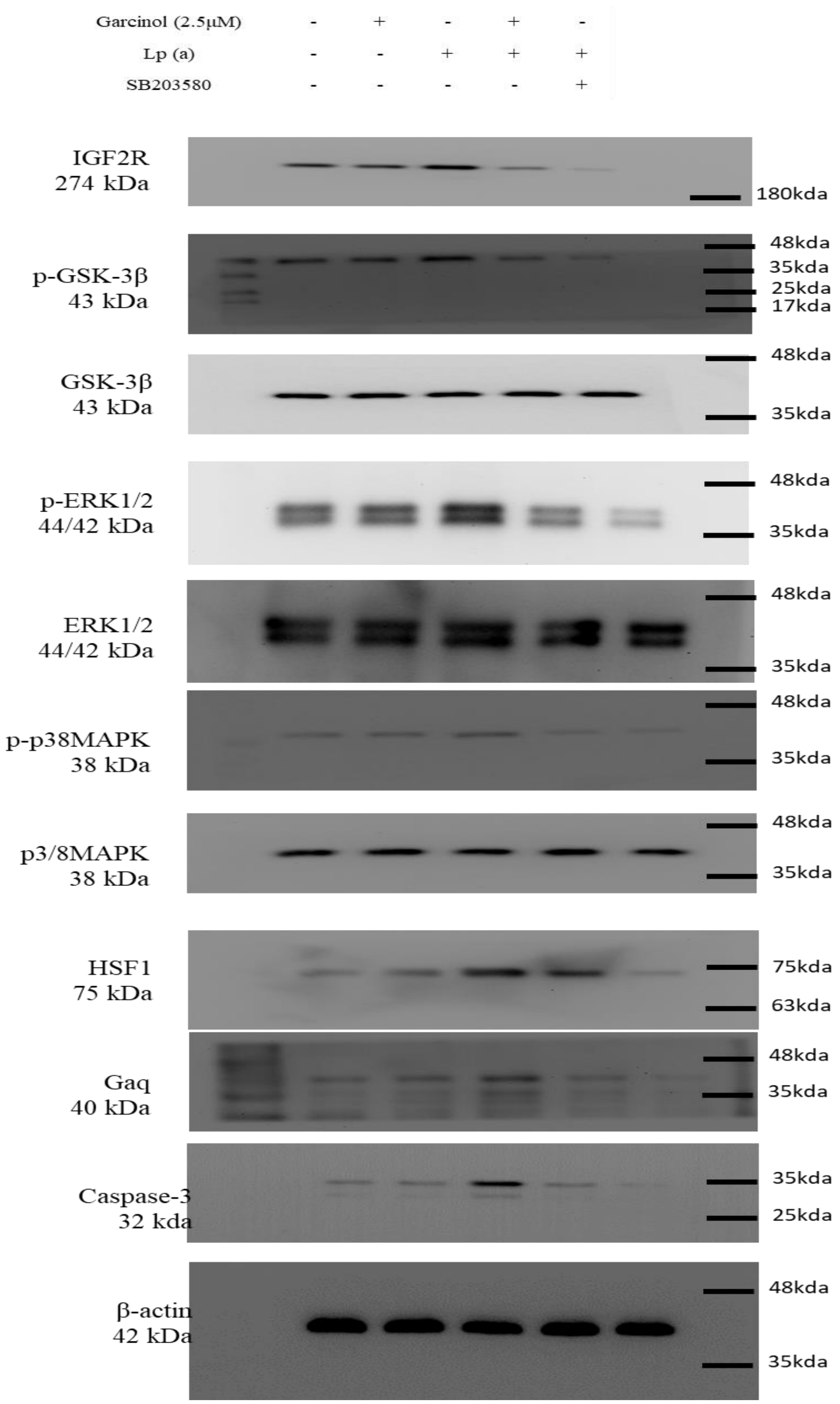

Supplementary Figure S5. Full-size blots of Figure 5A

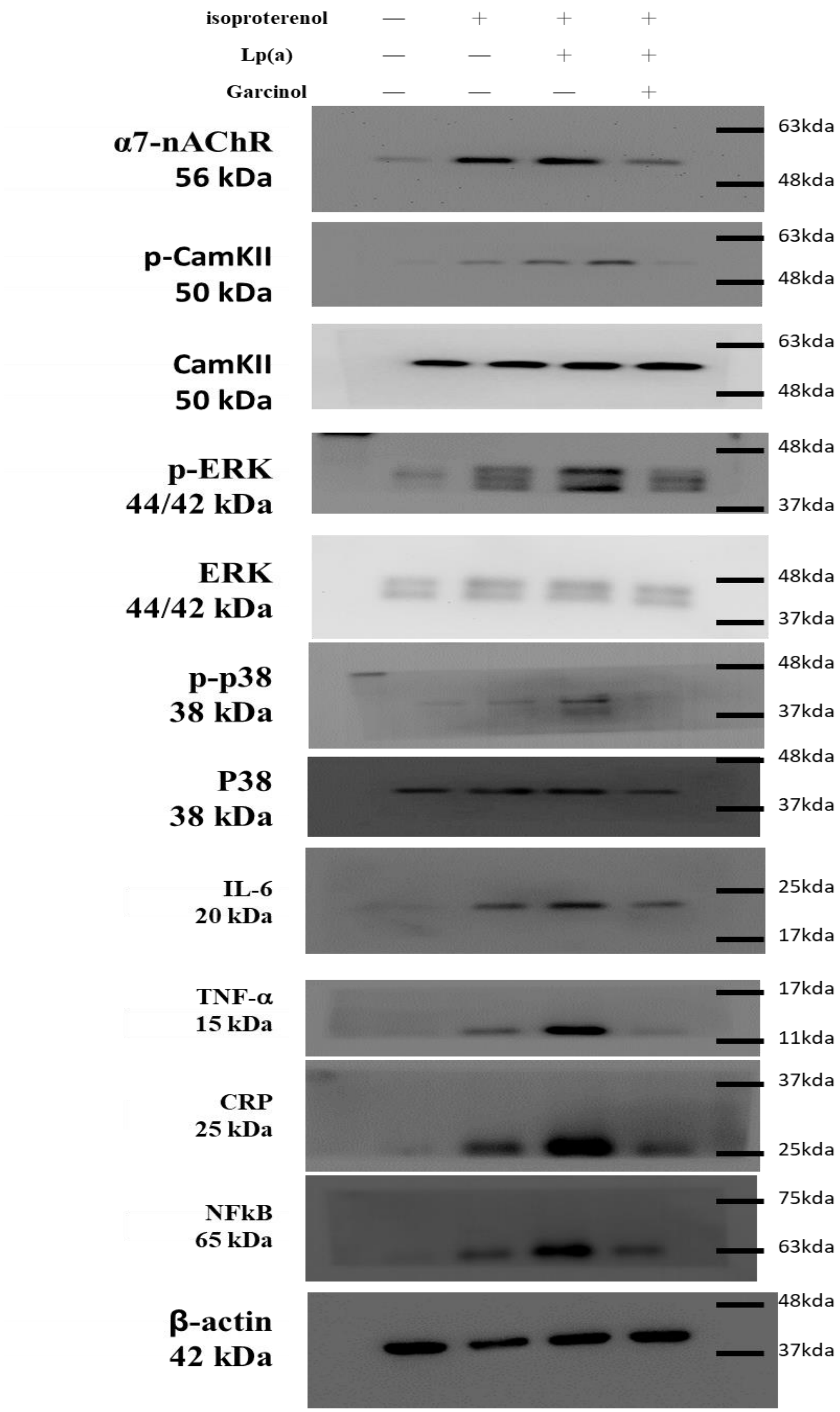

Supplementary Figure S6. Full-size blots of Figure 6D
